# Transcriptional profiling of circulating mononuclear cells from patients with chronic obstructive pulmonary disease receiving mesenchymal stromal cell infusions

**DOI:** 10.1002/sctm.21-0024

**Published:** 2021-08-18

**Authors:** Jesse D. Armitage, Dino B.A. Tan, Marian Sturm, Yuben P. Moodley

**Affiliations:** ^1^ Centre for Respiratory Health, School of Biomedical Sciences University of Western Australia Nedlands Western Australia Australia; ^2^ Stem Cell Unit Institute for Respiratory Health Nedlands Western Australia Australia; ^3^ Cell and Tissue Therapies WA Royal Perth Hospital Perth Western Australia Australia; ^4^ Regenerative Biology, Faculty of Health and Medical Science University of Western Australia Perth Western Australia Australia; ^5^ Department of Respiratory Medicine Fiona Stanley Hospital Murdoch Western Australia Australia

**Keywords:** clinical trials, gene expression, immunosuppression, mesenchymal stem cells

## Abstract

Chronic obstructive pulmonary disease (COPD) is an inflammatory airways disease with limited therapeutic options. We have previously shown that mesenchymal stromal cell (MSC) infusions are well tolerated in patients with COPD and reduce circulatory biomarkers associated with systemic inflammation and oxidative stress. This study aimed to delineate the underlying mechanisms further by characterizing the transcriptional networks in these patients and to explore the role of MSC‐derived paracrine factors in regulating these pathways. Allogeneic, bone marrow‐derived MSCs were systemically administered into patients with stable COPD (n = 9). Gene expression profiles from peripheral blood mononuclear cells (PBMCs) were analyzed across the first week after infusion. Paracrine mechanisms associated with these transcriptional changes were explored further by culturing patient PBMCs with MSC‐conditioned medium (MSC‐CM) or post‐MSC infusion (PI) plasma to measure the regulatory effects of soluble factors that may be derived from MSCs. MSC‐CM and PI‐plasma were characterized further to identify potential immunoregulatory candidates. MSC infusion elicited a strong but transient transcriptional response in patient PBMCs that was sustained up to 7 days. MSC infusion strongly downregulated transcriptional pathways related to interleukin (IL)‐8 and IL‐1β, which were also significantly inhibited in vitro following co‐culture of PBMCs with MSC‐CM and PI‐plasma. MSC‐derived soluble tumor necrosis factor receptor‐1, transforming growth factor‐β1, and extracellular vesicle‐associated microRNAs were identified as potential mechanisms promoting these changes, but depletion of these individual candidates revealed inconsistent results. MSC‐derived paracrine factors modulate important inflammatory pathways that are relevant to COPD pathogenesis. These data strengthen the hypothesis that therapies using MSCs and their secreted products may be beneficial to patients with COPD.

## Graphical abstract

Mesenchymal stromal cells (MSC) are potent immunomodulators that have shown promise in treating chronic obstructive pulmonary disease (COPD). Using transcriptomics, we have demonstrated that MSC infusions elicit a strong, yet transient (<7 days) transcriptional response in circulating mononuclear cells of COPD patients, attenuating key pro‐inflammatory pathways. These responses are likely mediated by soluble factors and/or products of MSC breakdown.

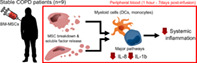

**Lessons learned**

- Mesenchymal stromal cells (MSCs) attenuate important pathogenic immunological pathways that underlie chronic airways disease.
- These effects were highly transient (waning by 7 days), suggesting that frequent, 1‐week doses may be important in achieving clinical benefit.
- MSC‐derived soluble factors may be responsible for these changes in vivo, suggesting new therapeutic options harnessing the MSC secretome.

**Significance statement**

Mesenchymal stromal cells are an emerging stem cell therapy that can be used to relieve chronic inflammation. As part of a phase I clinical study, this article reports for the first time that these stem cells modulate important pathological pathways that drive inflammation in patients with chronic airways disease. This study provides novel insights that may guide further investigation of mesenchymal stromal cell therapy for the treatment of patients with chronic airway disease.

## 1 INTRODUCTION

Chronic obstructive pulmonary disease (COPD) is among the top five causes of global morbidity and mortality. COPD is characterized by airway obstruction, inflammation, and irreversible lung remodeling.^1^ Systemic inflammation and oxidative stress are further features of COPD associated with comorbidities such as cardiovascular disease.^2^ Smoking is the commonest cause of COPD, although genetic predisposition and air pollution are other etiological factors. Despite optimal treatment and the cessation of precipitants (eg, smoking), chronic inflammation (evidenced by increased activation of T cells and elevation of pulmonary and systemic proinflammatory cytokines) and oxidative stress persist in most patients,^3^ highlighting the need to develop novel immunomodulatory strategies to address this condition.

Mesenchymal stromal cells (MSCs) are obtained from a variety of sources such as bone marrow and exert a range of anti‐inflammatory and reparative effects.^4^ These are largely mediated through paracrine mechanisms such as the release of anti‐inflammatory cytokines, growth factors, and extracellular vesicles.^5^ Hence, the use of MSCs in a broad range of inflammatory and immune‐mediated diseases is increasingly being investigated.^6^ Preclinical studies of MSCs in COPD demonstrate efficacy in alleviating inflammation and reducing emphysema following either systemic or intratracheal administration in rodent models; however, these findings have translated poorly in human studies.^7^ A large clinical trial, despite showing no significant clinical improvement in patients with stable COPD following a regimen of four monthly intravenous MSC infusions, demonstrated a reduction in circulating C‐reactive protein (CRP) after 1 month, suggesting that intravenous MSC infusion may alter systemic inflammation in patients with COPD.^8^ More recently, a trial using MSCs to minimize localized inflammation in response to one‐way endobronchial valve insertion in patients with COPD showed similar reductions in circulating CRP between 1 and 3 months after treatment.^9^

The reasons for a lack of efficacy in clinical trials to date are unclear, and this is underscored by a lack of mechanistic studies in humans. To delineate these mechanisms, we characterized the systemic immunological changes of patients with stable COPD (n = 9) receiving radiolabeled MSCs over 1 week. We previously showed short‐term reductions in levels of systemic proinflammatory (interleukin [IL]‐6, soluble [s]CD163) and oxidative stress (F2‐isoprostanes) biomarkers and increases in proportions of circulating regulatory T cells and monocytes across the first week after infusion.^10^ In the present study, we performed whole transcriptome sequencing of peripheral blood mononuclear cells (PBMCs) isolated from these patients with COPD before and after MSC infusion. These findings provide novel insights into the mechanisms of MSC‐mediated systemic immunomodulation, as these short‐term dynamics have not previously been described in human studies.

## 2 MATERIALS AND METHODS

### 2.1 Patient cohort and sample collection

A single‐site, phase I study (Australian clinical trials registry no. 12614000731695) was conducted to initially determine the effects of MSC infusion on clinical and safety endpoints in patients with stable COPD (described further in the supplemental online data). Nine patients with stable COPD received two doses of 2 × 10^6^ allogenic bone marrow‐derived MSCs per kilogram patient weight, 1 week apart.^10^ Eight patients received MSCs (both infusions) from one donor (female, 28 years old), whereas one patient received MSCs (both infusions) from a second donor (male, 20 years old). Patient demographics are provided in Table 1, and the study was approved by the ethics committee at Royal Perth Hospital (approval no. EC2012/103), where all patients had provided written informed consent. Heparinized venous peripheral blood was collected prior to MSC infusion (baseline) and 1 hour, 1 day, 2 days, and 7 days after the first infusion, followed by a collection 1 hour after the second infusion. Plasma was first isolated and stored at −80°C. PBMCs were isolated by Ficoll density gradient centrifugation and cryopreserved in liquid nitrogen.

**TABLE 1:** Baseline characteristic of the cohort

Characteristic	Cohort (n = 9)
Age, year	70 (62‐81)
BMI	30 (17‐47)
Male sex	4 [44%]
Medication use	
Salbutamol	9 [100%]
Salmeterol/fluticasone	7 [78%]
Tiotropium	7 [78%]
Lung‐related hospitalizations (3‐12 months before infusion)	4 [44%]
Steroid treatment required (prednisolone)	4
Lung function	
FEV_1_ (% predicted)	37 (23‐87)
FVC (% predicted)	80 (59‐106)
GOLD I (>80% pred. FEV_1_)	1 [11%]
GOLD II (50%‐80% pred. FEV_1_)	2 [22%]
GOLD III (30%‐50% pred. FEV_1_)	3 [33%]
GOLD IV (<30% pred. FEV_1_)	3 [33%]
Comorbidities	
Cardiovascular	5
Hypercholesterolemia	3
Depression and anxiety	2
Osteoporosis, osteoarthritis	1
Smoking status	
Ex‐smoker	9 [100%]
Years since cessation	15 (1‐36)
Pack/yr	32 (17‐59)

*Note*: Data are presented as median (range) or number of patients [percentage].

Abbreviations: BMI, body mass index; FEV_1_, forced expiratory volume in the first second; FVC, forced vital capacity; GOLD, Global Initiative for Chronic Obstructive Lung Disease.

### 2.2 RNA sequencing and analysis

Details on RNA extraction, sequencing, and preprocessing are described in the supplemental online data. Gene clustering was performed using the signed weighted gene coexpression network analysis (WGCNA) pipeline (Figure S1).^11^ Differential gene expression was performed using DESeq2. Differentially expressed genes (DEGs) were determined using the Wald chi‐squared test with a Benjamini‐Hochberg correction for false discovery rate (FDR; *q* value). DEGs with a value of *q* < .05 were considered statistically significant. The module eigengene was used as a measure of individual gene module expression. Overrepresented pathways and cell subset signatures within interesting modules were explored using Enrichr and ARCHS4 databases, respectively.^12^, ^13^ Significant overrepresentation is determined using Fisher's exact test with a Benjamini‐Hochberg correction for FDR. Identification of intramodular gene hubs was performed using a literature‐curated protein‐protein interaction database (Search Tool for the Retrieval of Interacting Genes/Proteins). Transcriptional networks were visualized using Cytoscape.

### 2.3 Quantitative polymerase chain reaction validation of whole transcriptome sequencing data

RNA from PBMC lysates was converted to cDNA using the SuperScript IV VILO kit (Thermo Fisher Scientific, Wilmington, Delaware) according to the manufacturer's instructions. All quantitative polymerase chain reactions (qPCRs) were performed using a Viia7 real‐time PCR system (Applied Biosystems, Waltham, Massachusetts). Data were recorded and analyzed using QuantStudio Real‐Time PCR software (Applied Biosystems), and normalization was performed against the expression of peptidyl‐prolyl cis‐trans isomerase as a housekeeping gene.

### 2.4 Harvesting of MSC‐conditioned medium

MSC‐conditioned medium (MSC‐CM) was collected after MSCs were cultured in 2% human serum albumin (HSA)/phenol red‐free (PRF) Dulbecco's modified Eagle's medium (DMEM) for 24 hours. MSC‐CM was centrifuged (450*g*, 10 minutes, 4°C) to remove cells, and cell‐free supernatant was passed through a 0.22 μm filter. MSC‐CM was concentrated 8‐fold by centrifugation (4000*g* for 90 minutes, 4°C) using a 3 kDa molecular weight cutoff (MWCO) filter (Merck Millipore, Burlington, Massachusetts). Plain medium containing 2% HSA/PRF‐DMEM was prepared and concentrated 8‐fold as the medium control.

### 2.5 Co‐culture of PBMCs with MSC‐CM and post‐MSC infusion plasma

Cryopreserved PBMCs were thawed, washed (450*g*, 10 minutes), and resuspended at 2 × 10^6^ cells per milliliter in 10% FCS/RPMI. Cells were seeded into a 96‐well round bottom tissue culture plate (200 000 cells per well). Cultures were set up in the absence of any stimulant (unstimulated) or with the addition of 0.1 μg/mL lipopolysaccharide (LPS; Sigma‐Aldrich, Castle Hill, New South Wales, Australia). Cultures were prepared in a final volume of 200 μL, containing 100 μL of cells with 100 μL of concentrated MSC‐CM or medium controls. For culturing with plasma, 50 μL of clarified patient plasma from baseline and 2 days and 7 days after infusion was co‐cultured with the corresponding patient's PBMC. Plasma was cultured in a final dilution of 1/10 in a total well volume of 200 μL. Final cultures were incubated at 37°C for 24 hours (5% CO_2_). Culture supernatants (150 μL) were then collected and stored at −80°C until further use.

### 2.6 Measurement of protein mediators

Quantification of proteins in MSC‐CM, patient plasma, and culture supernatants was performed using enzyme‐linked immunosorbent assays according to the manufacturer's instructions (tumor necrosis factor [TNF]‐α, interferon‐gamma [IFN‐γ], CCL18, IL‐10, IL‐5, and IP‐10 from BD Biosciences [San Jose, California]; IL‐8, soluble TNF receptor‐1 [sTNFR1], IL‐1β, and total transforming growth factor‐β1 [TGF‐β1] from R&D Systems [Minneapolis, MN]; and TNF‐α‐stimulated gene‐6 [TSG‐6] from RayBiotech [Norcross, Georgia]).

### 2.7 Isolation of MSC extracellular vesicles

Concentrated MSC‐CM (total 4 mL; 1 mL per column) was carefully applied to four qEV columns (Izon Sciences, Christchurch, New Zealand). Elution buffer (phosphate‐buffered saline) was sequentially added until 3 mL of total volume had passed through the column (fractions 1‐6 at 500 μL per fraction). Fractions 7‐10 (2 mL) were then collected from each column and combined to a final volume of 8 mL. Extracellular vesicle (EV)‐rich fractions were then concentrated using a 100 kDa MWCO filter at 4000*g* for 40 minutes at 4°C and topped up to 100 μL with phosphate‐buffered saline. Validation of MSC‐EVs was performed using nanoparticle tracking analysis and transmission electron microscopy (TEM) as previously described,^14^ in line with standard practices for EV characterization as outlined by Théry et al.^15^ Quantification of EVs after depletion (Figure S2) was performed using imaging flow cytometry as previously described.^14^

### 2.8 Quantification of plasma and MSC‐EV microRNAs

Extraction of microRNAs (miRNAs) from purified MSC‐EVs or patient plasma (pooled at each time point) was performed using the miRNeasy Kit (Qiagen, Hilden, Germany) according to the manufacturer's instructions. Reverse transcription and preamplification of cDNA was performed using TaqMan Advanced miRNA cDNA Synthesis kit (Thermo Fisher Scientific) according to the manufacturer's instructions. Preamplified cDNA was loaded on TaqMan Array Human MicroRNA cards (Thermo Fisher Scientific). Final qPCRs were performed using a Viia7 real‐time PCR system. Data were recorded and analyzed using QuantStudio Real‐Time PCR software. Raw miRNA expression was normalized using the global mean, as described by D'Haene et al.^16^

### 2.9 Statistical analysis

Except for the WGCNA pipeline and the calculation of DEGs, longitudinal assessments were performed using GraphPad Prism 5 software (GraphPad Software, San Diego, California). Wilcoxon signed‐ranked (nonparametric) tests were used to perform pairwise comparisons between time points with respect to baseline or control conditions. Changes with a value of *P* < .05 were considered statistically significant, and changes with marginal significance (*P* values between .05 and .1) are also noted.

## 3 RESULTS

### 3.1 MSC infusion promotes transient changes in gene expression within 7 days after infusion

**FIGURE 1:**
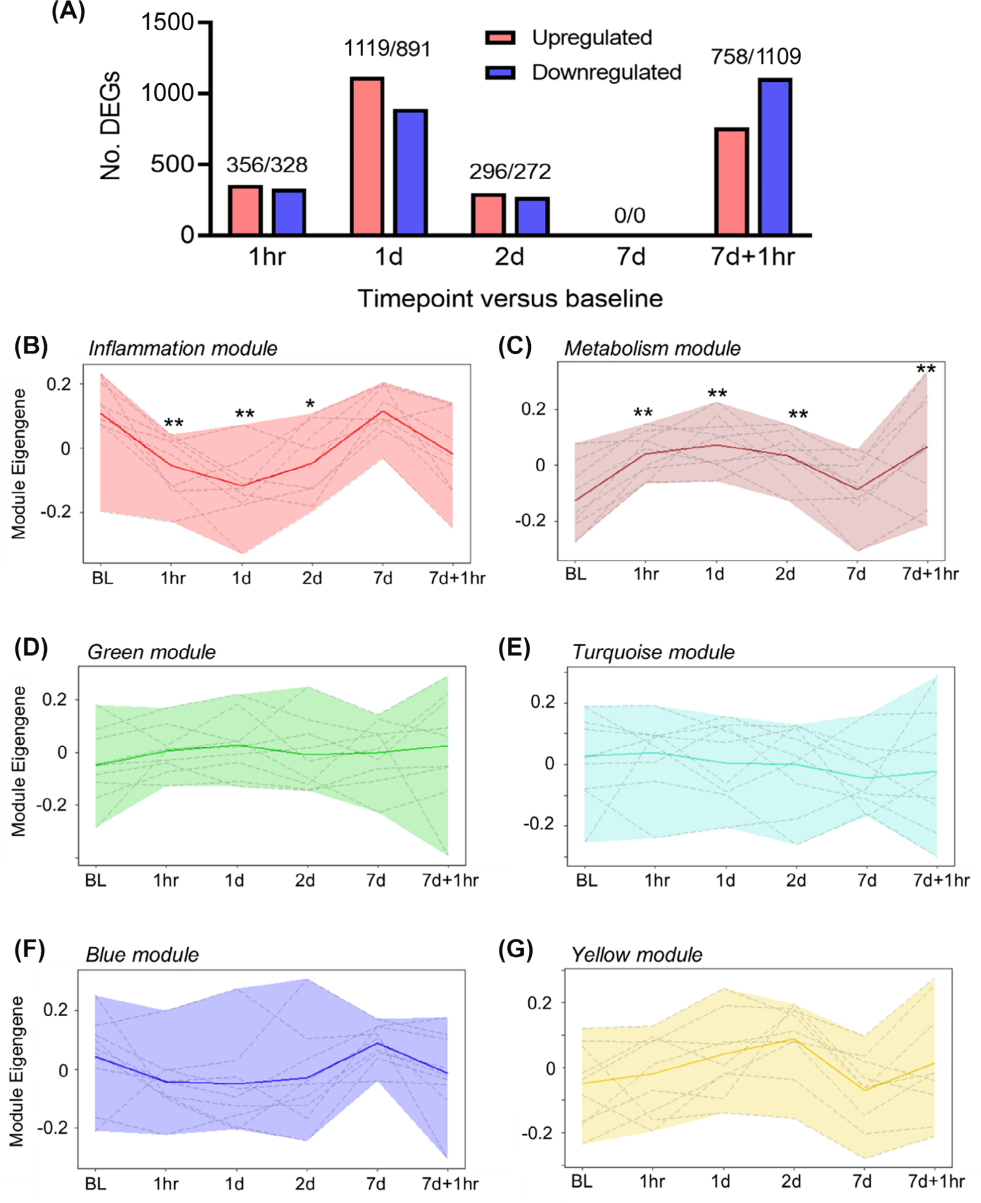
Weighted gene coexpression network analysis elucidates key gene modules that are significantly altered following mesenchymal stromal cell infusion. A, The total number of differentially expressed genes are shown for each comparison compared to baseline samples (upregulated in red; downregulated in blue, using a false discovery rate of q < 0.05). Module expression profiles for the inflammation, B, and metabolism modules, C, and the green, D, turquoise, E, blue, F, and yellow, G modules are visualized. Solid lines represent the average trend across the time course, with the dashed lines representing each patients' individual time course. **P < .01, *P < .5 (n = 9 patients). BL, baseline; DEG, differentially expressed gene

RNA sequencing was performed on mRNA extracted from patient PBMCs at baseline, 1 hour, and 1, 2, and 7 days after infusion and 1 hour after the second infusion. The number of DEGs (both up‐ and downregulated) peaked at 1 day after infusion and declined to no DEGs observed by 7 days after infusion. Following a second MSC infusion on the seventh day, a second spike in DEGs was observed after 1 hour (Figure 1A).

WGCNA generated six coexpression modules by unsupervised hierarchical clustering of coexpressing genes across all cohort samples (Figure S1). The red module was found to be enriched in inflammatory genes and hence termed the “inflammation” module, which was investigated further. The brown module was enriched in metabolic genes (hence termed the “metabolism” module) with no clear immunological function, and the other four modules (green, turquoise, blue, and yellow) were not enriched in any particular cellular or molecular processes and were not investigated further. Inflammation module expression (measured by the module eigengene) was significantly reduced between 1 hour and 2 days after infusion compared with baseline (Figure 1B). Conversely, there was a significant increase in the metabolism module expression between 1 hour and 2 days after infusion and 1 hour after the second infusion compared with baseline (Figure 1C). Green, turquoise, blue, and yellow module expression was unchanged across the time course (Figure 1C‐F).

### 3.2 MSC infusion attenuates proinflammatory transcriptional networks that are associated with COPD pathogenesis

**FIGURE 2:**
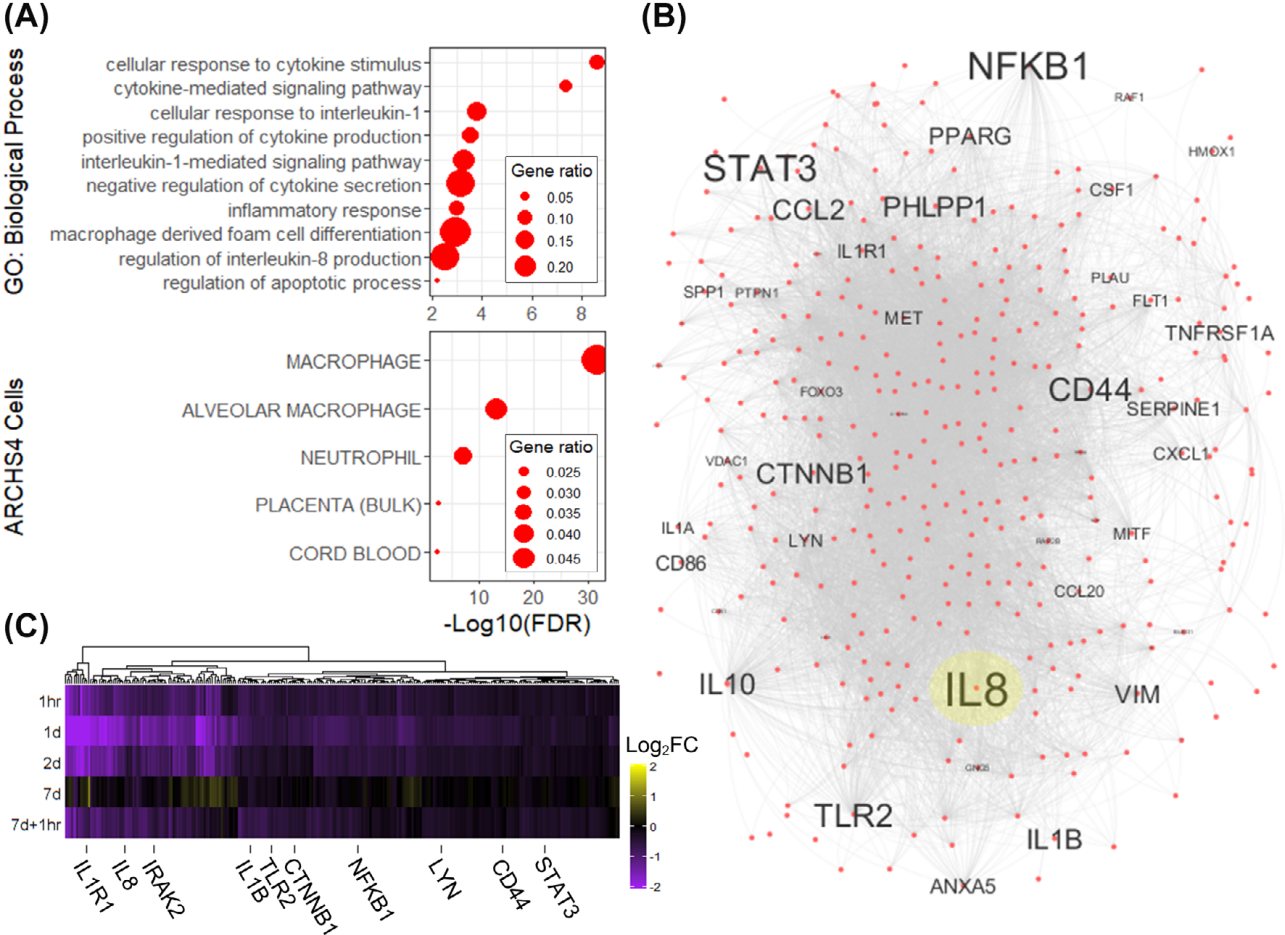
Modulation of inflammatory gene networks following mesenchymal stromal cell infusion. A, Overrepresented biological processes and cellular signatures from differentially expressed genes within the inflammation module. B, The transcriptional network was constructed using the Search Tool for the Retrieval of Interacting Genes/Proteins (STRING) database, and gene names with larger font indicate a higher combined weighted gene coexpression network analysis and STRING connectivity within the network. IL‐8, which is the central gene hub, is highlighted in yellow. C, Gene expression heatmap of inflammation module genes showing log_2_fold change compared with baseline. Specific genes that were validated by real‐time quantitative polymerase chain reaction are annotated (n = 9 patients). CTNNB1, catenin beta 1; FDR, false discovery rate; GO, gene ontology; IL, interleukin; IL1R1, interleukin‐1 receptor‐1; IRAK2, IL‐1 receptor‐associated kinase 2; LYN, Lyn kinase; NFKB1, nuclear factor kappa B subunit 1; STAT3, signal transducer and activator of transcription 3; TLR2, Toll‐like receptor 2

**FIGURE 3:**
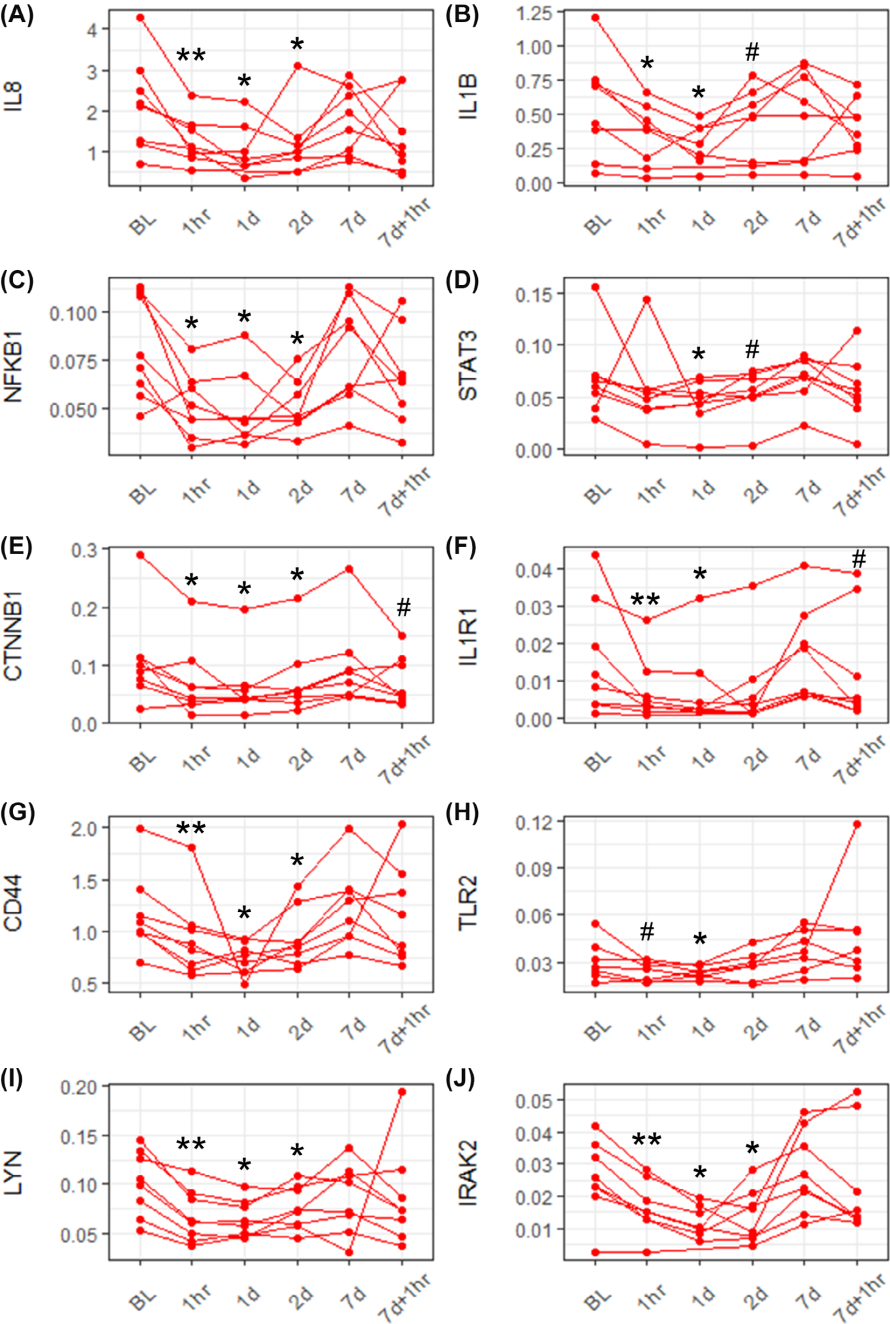
Validation of inflammation module genes by real‐time quantitative polymerase chain reaction. Peptidyl‐prolyl cis‐trans isomerase‐normalized gene expression of cytokines (IL‐8 and IL1‐B), A, B, transcription factors (NFKB1, STAT3, and CTNNB1), C, D, E, and signaling molecules (IL1R1, CD44, TLR2, LYN, and IRAK2), F, G, H, I, J. **P < .01, *P < .05, ^#^ *P* < .1 (n = 8 patients). BL, baseline; CTNNB1, catenin beta 1; IL, interleukin; IL1R1, interleukin‐1 receptor‐1; IRAK2, IL‐1 receptor‐associated kinase 2; LYN, Lyn kinase; NFKB1, nuclear factor kappa B subunit 1; STAT3, signal transducer and activator of transcription 3; TLR2, Toll‐like receptor 2

Cytokine signaling pathways, notably IL‐1 and IL‐8, were enriched in the inflammation module along with a strong macrophage/monocyte transcriptional signature (Figure 2A). The gene network of the inflammation module highlights IL‐8 as the central gene hub, along with other pro‐inflammatory genes, including nuclear factor kappa B subunit 1 (NFKB1), Toll‐like receptor 2 (TLR2), CD44, and signal transducer and activator of transcription 3 (STAT3) (Figure 2B). Consistent with its module eigengene levels, there is a common expression pattern for all genes within the inflammation module, where transcript levels show the strongest reduction around 1 day after infusion before returning to baseline levels by the seventh day (Figure 2C). Real‐time qPCR validation of mRNA encoding cytokines (IL‐8 and IL‐1B; Figure 3A,B), transcription factors (NFKB1, STAT3, and catenin beta 1; Figure 3C‐E), and signaling molecules (IL‐1 receptor‐1, CD44, TLR2, Lyn kinase, and IL‐1 receptor‐associated kinase 2; Figure 3F‐J) confirmed that the expression patterns of individual genes within this module were consistent with its corresponding module expression from the WGCNA pipeline.

### 3.3 MSC‐derived soluble factors contribute to the in vitro downregulation of IL‐8 and IL‐1b responses in patient PBMCs

**FIGURE 4:**
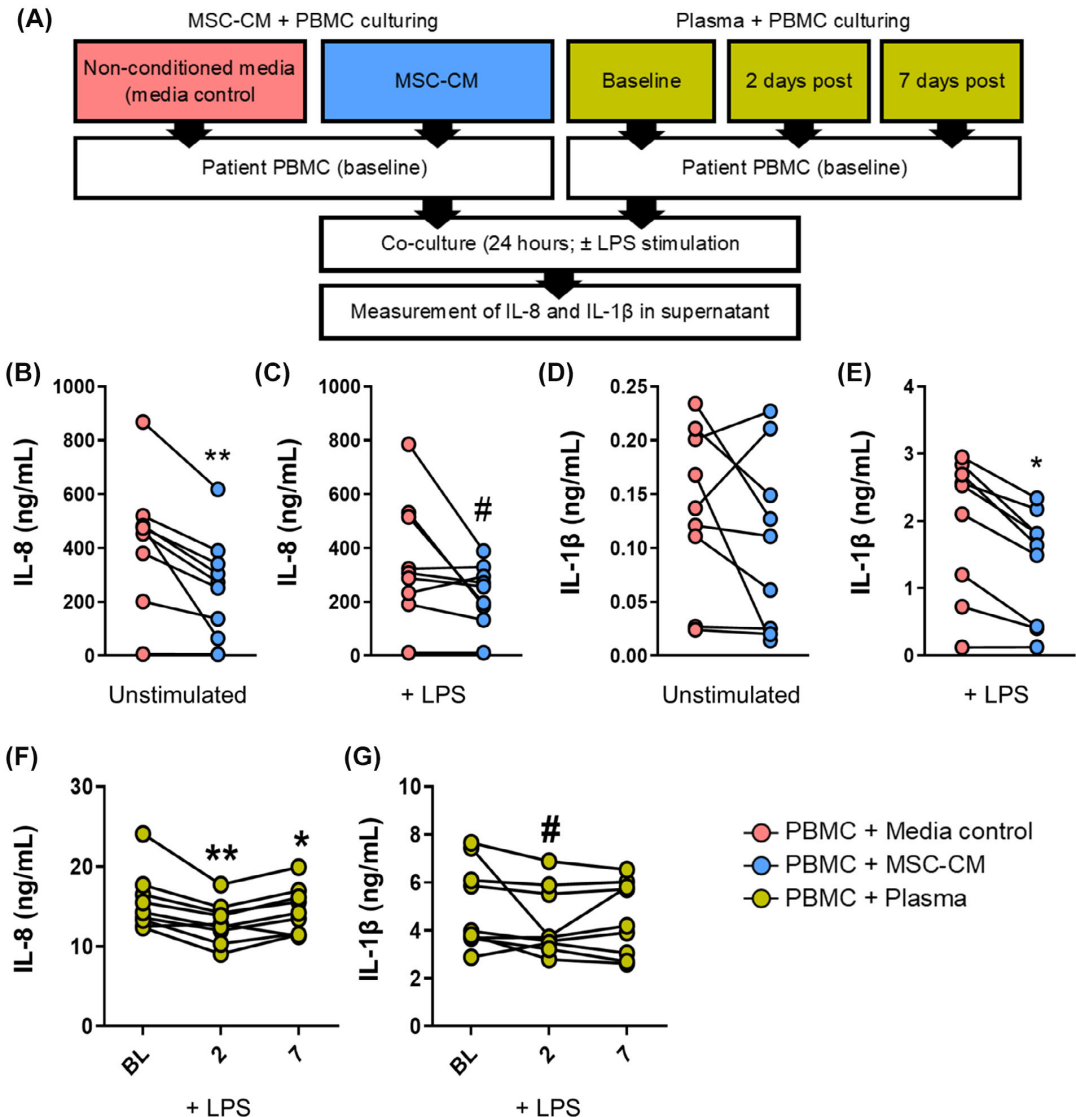
Mesenchymal stromal cell (MSC)‐derived soluble factors attenuate IL‐8 and IL‐1β cytokine production by PBMCs. A, A schematic depicts the experimental setup to evaluate the immunological effects of MSC‐derived soluble factors and soluble factors released into circulation following MSC infusion. B, C, IL‐8 production by PBMCs was measured under nonstimulated and LPS‐stimulated conditions in the presence of control medium and MSC‐CM. D, E, IL‐1β production by PBMCs was measured under nonstimulated and LPS‐stimulated conditions in the presence of control medium and MSC‐CM. F, G, IL‐8 and IL‐1β production by PBMCs was measured under LPS‐stimulated conditions in the presence of patient plasma collected before and after MSC infusion. All cytokine measurements were adjusted to account for background IL‐8 and IL‐1β that may be present in MSC‐CM and plasma. **P < .01, *P < .05, ^#^ *P* < .1 (n = 8 patients). BL, baseline; IL, interleukin; LPS, lipopolysaccharide; MSC‐CM, mesenchymal stromal cell‐conditioned medium; PBMC, peripheral blood mononuclear cell

Patient PBMCs were cultured in the presence of MSC‐CM or medium control to determine whether soluble factors directly produced by MSCs may downregulate IL‐1β and IL‐8 (Figure 4A). Treatment of PBMC with LPS resulted in significantly higher levels of IL‐8 and IL‐1β compared with unstimulated cultures (data not shown); however, levels of IL‐8 were not different in the presence of MSC‐CM/medium control and LPS compared with unstimulated PBMC (Figure 4A vs B). Compared with medium control, levels of IL‐8 were significantly reduced (*P* = .002) following culturing of MSC‐CM with nonstimulated PBMCs (Figure 4B), whereas LPS‐stimulated PBMCs showed a similar decreasing trend in IL‐8 (*P* = .06; Figure 4C). Levels of IL‐1β were unchanged in unstimulated conditions (Figure 4D); however, the MSC‐CM significantly reduced IL‐1β secretion by LPS‐stimulated PBMCs (*P* = .01; Figure 4E). Interestingly, the presence of MSC‐CM or control medium stimulated potent IL‐8 production, whereas IL‐1β was slightly reduced, suggesting that common components of the culture medium (ie, 1% HSA/DMEM) play a role in modulating cytokine production.

Culturing of PBMCs with patient post‐MSC infusion plasma (PI‐plasma) revealed that IL‐8 production was significantly attenuated in the presence of 2‐ and 7‐day PI‐plasma compared with baseline plasma (*P* = .08, *P* = .02; Figure 4C). Similarly, culturing with 2‐day PI‐plasma showed a reduction in IL‐1β compared with baseline plasma, although this was only marginally significant (*P* = .06; Figure 4F). Notably, the production of IL‐8 and IL‐1β by PBMC was greatly reduced in the presence of patient plasma (at any time points) compared with plasma‐free cultures, suggesting an abundance of inhibitory factors in plasma. Additional experiments also confirmed that PBMC viability remained unchanged with or without LPS stimulation following 24 hours of culture, demonstrating that viability is not a confounding variable affecting cytokine production (data not shown). Taken together, these findings suggest that common paracrine mechanisms found in MSC‐CM and PI‐plasma may inhibit IL‐8 and IL‐1β pathways.

### 3.4 Inhibition of IL‐8 and IL‐1β production by PBMC may be associated with high concentrations of anti‐inflammatory soluble factors in MSC‐CM

**FIGURE 5:**
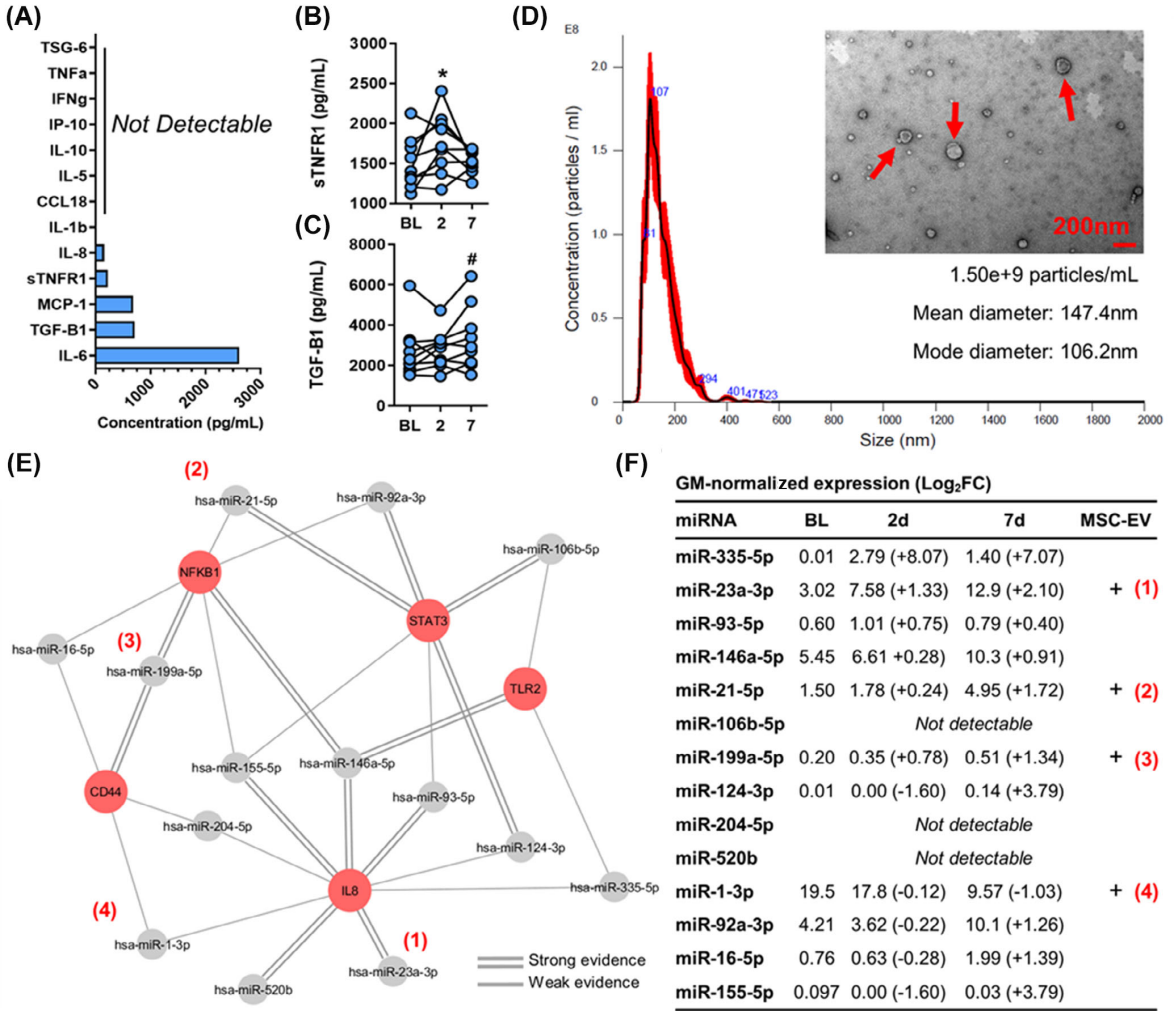
Identification of potential immunoregulatory mediators in mesenchymal stromal cell (MSC)‐conditioned medium (MSC‐CM) and plasma following MSC infusion. A, Quantification of soluble factors in MSC‐CM. B, C, Measurement of sTNFR1 (B) and TGF‐β1 (C) at baseline and 2 and 7 days after infusion. D, Characterization of MSC‐EVs by nanoparticle tracking analysis and transmission electron microscopy, where red arrows denote the presence of particles with EV‐like size and morphology. E, The miRNA‐gene network constructed using miRTarBase illustrates putative gene targets (red nodes) for each miRNA (grey nodes). F, Summary of global mean‐normalized miRNA expression in pooled patient plasma at baseline and 2 and 7 days after infusion (including their log_2_fold change) and their detectability in MSC‐EVs (+). Only the 14 miRNAs that target hub genes are visualized. The miRNAs found in MSC‐EVs and detectable in plasma are correspondingly annotated on the miRNA‐gene target network and the summary table. *P < .05, #*P* < .1 (n = 9 patients). 2d, day 2; 7d, day 7; BL, baseline; CCL18, chemokine (C‐C motif) ligand 18; EV, extracellular vesicle; FC, fold change; GM, global mean; hsa‐miR, homo sapiens‐microRNA; IFNg, interferon gamma; IL, interleukin; IP‐10, IFN‐γ inducible protein‐10; MCP‐1, monocyte chemoattractant protein‐1; miRNA, microRNA; sTNFR1, soluble tumor necrosis factor receptor‐1; TGF‐B1, transforming growth factor‐β1; TNFa, tumor necrosis factor‐α; TSG‐6, tumor necrosis factor‐α‐stimulated gene‐6

To identify potential factors that regulate IL‐1β and IL‐8, MSC‐CM was first screened for the presence of immunoregulatory proteins such as TNF‐α, TSG‐6, IFN‐γ, IFN‐γ inducible protein‐10 (IP‐10), IL‐10, IL‐5, chemokine (C‐C motif) ligand 18 (CCL18), IL‐1β, IL‐8, sTNFR1, monocyte chemoattractant protein‐1 (MCP‐1), TGF‐β1, and IL‐6. Two key anti‐inflammatory proteins, sTNFR1 and TGF‐β1, were detected in MSC‐CM (Figure 5A). Furthermore, sTNFR1 levels in plasma were significantly elevated 2 days after infusion (*P* = .02), whereas TGF‐β1 was elevated 7 days after infusion, although this was not significant (*P* = .07; Figure 5C).

To investigate the role of miRNA in regulating the gene hubs identified within the inflammation module, we also extracted miRNA from plasma after MSC infusion and from EVs isolated from MSC‐CM. Purified MSC‐EVs had an average diameter of 100‐150 nm, confirmed by nanoparticle tracking analysis, and were shown to have a morphology consistent with EVs by TEM (Figure 5D). Using miRTarBase (an online tool that maps putative miRNA‐target gene links), we identified 14 candidate miRNAs that target the top five gene hubs within the inflammation module (IL‐8, CD44, NFKB1, STAT3, and TLR2; Figure 5E). Of these 14 candidates, 11 were detectable in plasmas samples from our cohort, and four were also detectable in MSC‐EVs (Figure 5F). To delineate the role of MSC‐EVs, TGF‐β1, and sTNFR1 in attenuating IL‐8, we co‐cultured patient PBMCs with MSC‐CM that was depleted for these mediators; however, we observed no significant changes in IL‐8 expression compared with PBMCs cultured with undepleted MSC‐CM (Figure S2).

## 4 DISCUSSION

The present study describes changes in PBMC gene expression profiles following MSC infusion, using unbiased correlation networks to identify novel pathways of immunomodulation. We also describe that these pathways can be potentially attenuated by MSC‐derived paracrine factors in vitro. In the same cohort of patients, our previous work has characterized the biodistribution of intravenously infused MSCs, where there is an early breakdown of MSCs (within 7 days) that are subsequently taken up by the reticuloendothelial system.^10^ This rapid loss of MSCs is likely to impact the longevity of the beneficial effects of MSC infusion. Nevertheless, numerous preclinical studies in COPD have demonstrated that MSC infusions can successfully attenuate inflammation and repair damaged lung tissue.^7^ Despite this, results from clinical trials in COPD have been disappointing,^8^, ^9^ highlighting the need to better understand these mechanisms in patients with COPD.

In this study, the transcriptional response in PBMCs to MSC infusion was shown to be potent yet transient, with a lack of DEGs observed by 7 days after infusion. This is in line with a recent phase I pilot study in patients with septic shock receiving MSC infusions, where plasma inflammatory cytokine levels (IL‐1β, IL‐8, IL‐6, MCP‐1, and IL‐2) are most prominently reduced around 12‐24 hours after infusion before reverting to baseline levels in the subsequent days.^17^ These effects are likely a consequence of the rapid breakdown of MSCs following intravenous infusion.

Network analysis reveals that genes within the downregulated module were associated with inflammatory cytokines pathways such as IL‐8 and IL‐1β. These pathways are especially important in COPD pathogenesis, as elevated levels of IL‐8 and IL‐1β promote chronic inflammation and decline in pulmonary function.^18^, ^19^ Both these cytokines are potent proinflammatory mediators that contribute to systemic and airway inflammation; however, IL‐8 in particular is a major mediator of neutrophil activation and chemotaxis, leading to a high oxidative stress burden and infiltration of neutrophils into the lungs.^20^ These findings are in line with our previous work, where MSC infusion reduced levels of circulating proinflammatory and oxidative stress biomarkers.^10^ Other work has also shown that MSCs can strongly attenuate respiratory burst in neutrophils, either directly or through the actions of mononuclear cells preconditioned by MSCs such as regulatory T cells.^21^, ^22^, ^23^ Current medications such as corticosteroids remain ineffective at targeting this axis of COPD pathogenesis,^24^ and clinical trials using biologics against both IL‐1β and IL‐8 have lacked efficacy or failed to achieve safety outcomes.^25^, ^26^ Moreover, these treatment options have little effect on limiting the extensive and irreversible lung damage triggered by viral or bacterial acute exacerbations, which are a major cause of morbidity and mortality in patients with COPD. Our data indicate that MSC infusions, due to their short‐term and transient effects, may not be useful in alleviating chronic inflammation in the long term. However, a recent retrospective analysis by Weiss et al showed that infusion of MSCs into patients with COPD with elevated baseline CRP (as a marker of systemic inflammation) significantly reduced long‐term (>3 months) systemic inflammation and improved pulmonary function.^27^ This suggests that MSC infusion may have long‐term benefits for patients with higher systemic inflammation. Due to the small cohort size in our study, we were unable to determine any significant differences in treatment response among those with high or low baseline systemic inflammation. Combined with findings from our previous work demonstrating that MSC infusions were safe and well tolerated by all patients,^10^ this study suggests that MSC infusions may be a useful alternative in targeting neutrophilic inflammation.

Paracrine factors produced by MSCs are known to be key drivers of immunomodulation. We explored the role of MSC‐derived soluble factors in attenuating IL‐8 and IL‐1β. In vitro co‐cultures containing MSC‐CM suppressed IL‐8 and IL‐1β production by PBMCs. Moreover, we showed that PI‐plasma was also capable of suppressing the production of these cytokines, suggesting these same MSC‐derived soluble factors may potentially be released systemically following MSC infusion. Although it is plausible that these changes may reflect a reduction in circulating proinflammatory mediators that dampen PBMC cytokine production, we identified common molecular drivers such as sTNFR1 and TGF‐β1 that are produced by MSCs and elevated in plasma following MSC infusion. Indeed, sTNFR1 and TGF‐β1 are well described anti‐inflammatory paracrine mediators produced by MSCs.^28^, ^29^, ^30^, ^31^ Additional in vitro experiments confirmed that patient PBMC isolated from day 2 or 7 after infusion did not significantly increase sTNFR1 and TGF‐B1 production compared with baseline PBMC, suggesting that these changes in plasma are more likely driven directly by MSCs (data not shown). Of note, MSC‐CM also contained high amounts of IL‐6, which is a well‐known pleiotropic cytokine that has been shown to downregulate IL‐1 and TNF activity in specific models.^32^, ^33^ Although we have not explored the effects of IL‐6 in our study, further investigation is warranted given that IL‐6 has been known to regulate other MSC paracrine mechanisms such as prostaglandin E2 secretion.^34^

Our study also explored the EV compartment of the MSC secretome, as EV cargo such as miRNAs are known to regulate immune responses.^35^ We discovered a panel of miRNAs that may be responsible for the inhibition of IL‐8 and IL‐1β, including miR‐23a‐3p, miR‐21‐5p, and miR‐199a‐5p, which were elevated in plasma following MSC infusion and detectable in MSC‐EVs. Notably, these miRNAs are known to exert immunoregulatory functions.^36^, ^37^, ^38^ Another miRNA of interest is miR‐335‐5p, which showed the highest increase in plasma after infusion. Although our experiments could not detect miR‐335‐5p in MSC‐EVs, there is evidence of its presence in MSCs.^39^ This miRNA may be important in COPD pathogenesis, as it has been demonstrated that miR‐335‐5p is strongly downregulated in PBMCs from patients with COPD and severe emphysema.^40^ Collectively, we have described several miRNAs that may play a role in attenuating IL‐8 and IL‐1β pathways and should be investigated in future studies. Intriguingly, PBMC cultures containing MSC‐CM that were individually depleted for sTNFR1, TGF‐β1, and MSC‐EVs showed no consistent effect on IL‐8 expression (Figure S2). Although different mechanisms may be acting in each patient, it is also plausible that these candidates act in concert to attenuate these inflammatory pathways, highlighting the therapeutic potential of “whole” MSC‐CM rather than its individual constituents. Moreover, although we speculate that changes in concentrations of inflammatory mediators in plasma alter the inflammatory response of PBMCs, more mechanistic studies are required to determine whether PBMCs are the cause or effect of these changes in plasma composition.

## 5 CONCLUSION

We show that MSC infusion downregulated several pathogenically relevant transcriptional pathways in patients with stable COPD. Notably, the variability of responses was not attributed to MSC donor type, mainly due to most patients (n = 8) receiving MSCs from a single donor. Our exploratory study has also outlined several potential paracrine mechanisms that may be exerting these effects, demonstrating the therapeutic potential of MSC‐CM. The insights gained from this study warrants further investigation of MSCs and/or their secreted factors as a novel therapeutic intervention in chronic airways diseases.

## CONFLICT OF INTEREST

M.S. declared outside the submitted work for Isopogen Pty Ltd. and a patent PCT/AU2014/001031 licensed to Cell & Tissue Therapies WA. The other authors declared no potential conflicts of interest.

## author contributions

J.D.A.: conception/design, collection and/or assembly of data, data analysis and interpretation, manuscript writing; D.B.A.T.: conception/design, collection and/or assembly of data, data analysis and interpretation, final approval of manuscript; M.S.: conception/design, provision of study material or patients, data analysis and interpretation, final approval of manuscript; Y.P.M.: conception/design, financial support, administrative support, data analysis and interpretation, provision of study material or patients, final approval of manuscript.

## Supporting information


**Data S1.** Supporting information.

## Data Availability

Additional information, including Raw RNAseq counts and differential gene expression outputs, are provided in the online supplementary material. Additional data can be obtained from the corresponding author upon reasonable request.

## References

[1] Barbu C , Iordache M , Man MG . Inflammation in COPD: pathogenesis, local and systemic effects. Rom J Morphol Embryol. 2011;52:21‐27. 21424028

[2] Cavaillès A , Brinchault‐Rabin G , Dixmier A , et al. Comorbidities of COPD. Eur Respir Rev. 2013;22:454‐475. 24293462 10.1183/09059180.00008612 PMC9639181

[3] Mortaz E , Masjedi MR , Rahman I . Outcome of smoking cessation on airway remodeling and pulmonary inflammation in COPD patients. Tanaffos. 2011;10:7‐11. PMC4153156 25191369

[4] Ma S , Xie N , Li W , et al. Immunobiology of mesenchymal stem cells. Cell Death Differ. 2014;21:216‐225. 24185619 10.1038/cdd.2013.158 PMC3890955

[5] Yagi H , Soto‐Gutierrez A , Parekkadan B , et al. Mesenchymal stem cells: mechanisms of immunomodulation and homing. Cell Transplant. 2010;19:667‐679. 20525442 10.3727/096368910X508762 PMC2957533

[6] Kim N , Cho SG . Clinical applications of mesenchymal stem cells. Korean J Intern Med. 2013;28:387‐402. 23864795 10.3904/kjim.2013.28.4.387 PMC3712145

[7] Broekman W , Khedoe PPSJ , Schepers K , et al. Mesenchymal stromal cells: a novel therapy for the treatment of chronic obstructive pulmonary disease? Thorax. 2018;73:565‐574. 29653970 10.1136/thoraxjnl-2017-210672 PMC5969341

[8] Weiss DJ , Casaburi R , Flannery R , et al. A placebo‐controlled, randomized trial of mesenchymal stem cells in COPD. Chest. 2013;143:1590‐1598. 23172272 10.1378/chest.12-2094 PMC4694112

[9] de Oliveira HG , Cruz FF , Antunes MA , et al. Combined bone marrow‐derived mesenchymal stromal cell therapy and one‐way endobronchial valve placement in patients with pulmonary emphysema: a phase I clinical trial. stem cells translational med. 2017;6:962‐969. 10.1002/sctm.16-0315 PMC5442791 28186686

[10] Armitage J , Tan D , Troedson R , et al. Mesenchymal stromal cell infusion modulates systemic immunological responses in stable COPD patients: a phase I pilot study. Eur Respir J. 2018;51:1702369. 29348155 10.1183/13993003.02369-2017

[11] Langfelder P , Horvath S . WGCNA: an R package for weighted correlation network analysis. BMC Bioinformatics. 2008;9:559. 19114008 10.1186/1471-2105-9-559 PMC2631488

[12] Kuleshov MV , Jones MR , Rouillard AD , et al. Enrichr: a comprehensive gene set enrichment analysis web server 2016 update. Nucleic Acids Res. 2016;44:W90‐W97. 27141961 10.1093/nar/gkw377 PMC4987924

[13] Lachmann A , Torre D , Keenan AB , et al. Massive mining of publicly available RNA‐seq data from human and mouse. Nat Commun. 2018;9:1366. 29636450 10.1038/s41467-018-03751-6 PMC5893633

[14] Armitage JD , Tan DBA , Cha L , et al. A standardised protocol for the evaluation of small extracellular vesicles in plasma by imaging flow cytometry. J Immunol Methods. 2019;468:61‐66. 30885719 10.1016/j.jim.2019.03.006

[15] Théry C , Witwer KW , Aikawa E , et al. Minimal information for studies of extracellular vesicles 2018 (MISEV2018): a position statement of the International Society for Extracellular Vesicles and update of the MISEV2014 guidelines. J Extracell Vesicles. 2018;7:1535750. 30637094 10.1080/20013078.2018.1535750 PMC6322352

[16] D'Haene B , Mestdagh P , Hellemans J , et al. miRNA expression profiling: from reference genes to global mean normalization. Methods Mol Biol. 2012;822:261‐272. 22144205 10.1007/978-1-61779-427-8_18

[17] Schlosser K , Wang JP , Dos Santos C , et al. Effects of mesenchymal stem cell treatment on systemic cytokine levels in a phase 1 dose escalation safety trial of septic shock patients. Crit Care Med. 2019;47:918‐925. 30720538 10.1097/CCM.0000000000003657 PMC6629173

[18] Agustí A , Edwards LD , Rennard SI , et al. Persistent systemic inflammation is associated with poor clinical outcomes in COPD: a novel phenotype. PLoS One. 2012;7:e37483. 22624038 10.1371/journal.pone.0037483 PMC3356313

[19] Zemans RL , Jacobson S , Keene J , et al. Multiple biomarkers predict disease severity, progression and mortality in COPD. Respir Res. 2017;18:117. 28610627 10.1186/s12931-017-0597-7 PMC5470282

[20] Di Stefano A , Capelli A , Donner CF . Role of interleukin‐8 in the pathogenesis and treatment of COPD. Chest. 2004;126:676‐678. 15364741 10.1378/chest.126.3.676

[21] Chen QH , Wu F , Liu L , et al. Mesenchymal stem cells regulate the Th17/Treg cell balance partly through hepatocyte growth factor in vitro. Stem Cell Res Ther. 2020;11:91. 32111238 10.1186/s13287-020-01612-y PMC7049226

[22] Espinosa G , Plaza A , Schenffeldt A , et al. Equine bone marrow‐derived mesenchymal stromal cells inhibit reactive oxygen species production by neutrophils. Vet Immunol Immunopathol. 2020;221:109975. 32087476 10.1016/j.vetimm.2019.109975

[23] Jung YJ , Park YY , Huh JW , et al. The effect of human adipose‐derived stem cells on lipopolysaccharide‐induced acute respiratory distress syndrome in mice. Ann Transl Med. 2019;7:674. 31930075 10.21037/atm.2019.10.48 PMC6944600

[24] Barnes PJ . Inflammatory endotypes in COPD. Allergy. 2019;74:1249‐1256. 30834543 10.1111/all.13760

[25] Mahler DA , Huang S , Tabrizi M , et al. Efficacy and safety of a monoclonal antibody recognizing interleukin‐8 in COPD: a pilot study. Chest. 2004;126:926‐934. 15364775 10.1378/chest.126.3.926

[26] Calverley PMA , Sethi S , Dawson M , et al. A randomised, placebo‐controlled trial of anti–interleukin‐1 receptor 1 monoclonal antibody MEDI8968 in chronic obstructive pulmonary disease. Respir Res. 2017;18:153. 28793896 10.1186/s12931-017-0633-7 PMC5551010

[27] Weiss DJ , Segal K , Casaburi R , et al. Effect of mesenchymal stromal cell infusions on lung function in COPD patients with high CRP levels. Respir Res. 2021;22:142. 33964910 10.1186/s12931-021-01734-8 PMC8106850

[28] Yagi H , Soto‐Gutierrez A , Navarro‐Alvarez N , et al. Reactive bone marrow stromal cells attenuate systemic inflammation via sTNFR1. Mol Ther. 2010;18:1857‐1864. 20664529 10.1038/mt.2010.155 PMC2951565

[29] Liu F , Qiu H , Xue M , et al. MSC‐secreted TGF‐β regulates lipopolysaccharide‐stimulated macrophage M2‐like polarization via the Akt/FoxO1 pathway. Stem Cell Res Ther. 2019;10:345. 31771622 10.1186/s13287-019-1447-y PMC6878630

[30] Martire A , Bedada FB , Uchida S , et al. Mesenchymal stem cells attenuate inflammatory processes in the heart and lung via inhibition of TNF signaling. Basic Res Cardiol. 2016;111:54. 27435289 10.1007/s00395-016-0573-2 PMC4951509

[31] de Araújo FV , Carrillo‐Gálvez AB , Martín F , et al. TGF‐β and mesenchymal stromal cells in regenerative medicine, autoimmunity and cancer. Cytokine Growth Factor Rev. 2018;43:25‐37. 29954665 10.1016/j.cytogfr.2018.06.002

[32] Xing Z , Gauldie J , Cox G , et al. IL‐6 is an antiinflammatory cytokine required for controlling local or systemic acute inflammatory responses. J Clin Invest. 1998;101:311‐320. 9435302 10.1172/JCI1368 PMC508569

[33] Murakami M , Kamimura D , Hirano T . Pleiotropy and specificity: insights from the interleukin 6 family of cytokines. Immunity. 2019;50:812‐831. 30995501 10.1016/j.immuni.2019.03.027

[34] Bouffi C , Bony C , Courties G , et al. IL‐6‐dependent PGE2 secretion by mesenchymal stem cells inhibits local inflammation in experimental arthritis. PLoS One. 2010;5:e14247. 21151872 10.1371/journal.pone.0014247 PMC2998425

[35] Ferguson SW , Wang J , Lee CJ , et al. The microRNA regulatory landscape of MSC‐derived exosomes: a systems view. Sci Rep. 2018;8:1419. 29362496 10.1038/s41598-018-19581-x PMC5780426

[36] Qu JQ , Yi HM , Ye X , et al. miR‐23a sensitizes nasopharyngeal carcinoma to irradiation by targeting IL‐8/Stat3 pathway. Oncotarget. 2015;6:28341‐28356. 26314966 10.18632/oncotarget.5117 PMC4695064

[37] Dai L , Gu L , Di W . miR‐199a attenuates endometrial stromal cell invasiveness through suppression of the IKKβ/NF‐κB pathway and reduced interleukin‐8 expression. Mol Hum Reprod. 2012;18:136‐145. 21989168 10.1093/molehr/gar066 PMC3292395

[38] Reis M , Mavin E , Nicholson L , et al. Mesenchymal stromal cell‐derived extracellular vesicles attenuate dendritic cell maturation and function. Front Immunol. 2018;9:2538. 30473695 10.3389/fimmu.2018.02538 PMC6237916

[39] Clark EA , Kalomoiris S , Nolta JA , et al. Concise review: microRNA function in multipotent mesenchymal stromal cells. stem cells. 2014;32:1074‐1082. 24860868 10.1002/stem.1623 PMC10668871

[40] Esquinas C , Janciauskiene S , Gonzalo R , et al. Gene and miRNA expression profiles in PBMCs from patients with severe and mild emphysema and PiZZ alpha1‐antitrypsin deficiency. Int J Chron Obstruct Pulmon Dis. 2017;12:3381‐3390. 29238183 10.2147/COPD.S145445 PMC5713702

