# Supplementary material for: Transcriptional profiling of circulating mononuclear cells from patients with chronic obstructive pulmonary disease receiving mesenchymal stromal cell infusions
Source: Stem Cells Transl Med. 2021 Aug 18;10(11):1470–81. doi: 10.1002/sctm.21-0024 (PMC8550696; doi:10.1002/sctm.21-0024)
Supplement: Supplementary file 1 — Data S1. Supporting information. [file SCT3-10-1470-s001.docx]

**Transcriptional profiling of circulating mononuclear cells from COPD patients receiving mesenchymal stromal cell infusions**

Armitage J.D.^1, 2^, Tan D.B.A.^1,2,3^, Sturm M.^3,4^, Moodley Y.P^1,2,5^

^1^Centre for Respiratory Health, School of Biomedical Sciences, University of Western Australia, Nedlands, WA, Australia. ^2^Stem Cell Unit, Institute for Respiratory Health, Nedlands, WA, Australia ^3^Cell and Tissue Therapies WA, Royal Perth Hospital, Perth, Australia. ^4^Regenerative Biology, Faculty of Health and Medical Science, University of Western Australia, Perth, Australia. ^5^Department of Respiratory Medicine, Fiona Stanley Hospital, Murdoch, WA, Australia.

**Corresponding author:**

Yuben P. Moodley

School of Medicine, University of Western Australia,

Level 2, Harry Perkins Institute of Medical Research,

Fiona Stanley Hospital Campus,

5 Robin Warren Drive, Murdoch, Perth, WA 6150

**Email:** [yuben.moodley@uwa.edu.au](mailto:yuben.moodley@uwa.edu.au)

**Supplementary Methods**

**Isolation, expansion and characterisation of MSCs**

Human bone marrow-derived MSCs were manufactured under good manufacturing practise (GMP) conditions (Therapeutic Goods Administration Licence No: MI-25112004-LI-000212-1) at the Cell and Tissue Therapies Western Australia (CTTWA) based at the Royal Perth Hospital (RPH). Bone marrow (10mL) was aspirated from unrelated normal volunteers, medically assessed as suitable and demonstrating negative testing for infectious disease markers (HIV-1/-2, HCV, HBV, HTLV-1/-2 and syphilis). MSC protocols for preparation and administration were followed as previously described ^1^. MSCs were isolated from marrow mononuclear fractions by adherence to tissue culture plastic and culture expanded up to passage 5. Passage 5 cells were cryopreserved in 10% dimethyl sulfoxide 50% PlasmaLyte (Baxter healthcare, Norfolk, UK), 20% normal saline, and 20% human serum albumin (HSA; Commonwealth Serum Laboratories, Victoria, AU) in aliquots of 50-100x10^6^ cells and stored in liquid nitrogen. Before release for clinical administration, MSC products were required to have cell viability greater than 70%, negative microbial contamination testing and demonstrate MSC immunophenotype (positivity for CD90, CD73 and CD105, whilst negative for HLA-DR, CD45, CD11b and CD19) and tri-lineage differentiation capacity post-thawing.

**Extraction of RNA, sequencing, and pre-processing of RNAseq data**

RNA was extracted from PBMC lysates using the RNeasy^®^ MinElute^®^ Cleanup Kit (Qiagen, Hilden, Germany) according to manufacturer’s instructions. Purified mRNA was converted into cDNA libraries using the TruSeq Stranded mRNA Library Preparation Kit (Illumina, Scoresby, VIC, AU) according to manufacturer’s instructions. Samples were sequenced on a HiSeq2500 (Illumina, Scoresby, VIC, AU; 50-bp single end reads, ∼19 million reads per sample) at the Australian Genome Research Facility (AGRF, Perth, AU). Raw files were processed by FastQC and aligned to the human genome by HISAT2. Read quantitation and post-alignment QC was performed using SummerizeOverlaps and SAMSat respectively. Gene counts were filtered and normalised by variance stabilising transformation (VST). Batch effects associated with sequencing lane were corrected for using the ComBat algorithm ^2^.

**Systemic delivery of MSCs into patients**

Patients received two infusions of approximately 2x10^6^ MSCs/kg a week apart (mean 1.8x10^6^/kg, range 0.9-2x10^6^/kg), the first infusion being ^111^Indium labelled cells ^3^ and the second infusion being unlabelled cells. MSCs for the study were manufactured from 2 donors, a male aged 19 years and a female aged 17 years. Patients only received cells from a single donor for both infusion episodes. The cell dosage, frequency and total number of infusions were based on that previously utilised in studies at CTTWA for systemic MSC administration in graft versus host and Crohns’ disease as representative chronic inflammatory diseases and met criteria in accordance with the international society for cellular therapy ^1 4 5^.

**Depletion of mediators from MSC-CM and validation**

Streptavidin-coated magnetic beads (100μg; Thermofisher, Wilmington, DE) were first coupled with either biotinylated anti-TNFR1 (30μg; Thermofisher), anti-TGF-β1 (30μg, targeting the latency-associated peptide; BioLegend, San Diego, CA, USA) or an anti-tetraspanin cocktail consisting of anti-CD9 (10μg; BioLegend), anti-CD63 (10μg; BioLegend) and anti-CD81 (10μg; BioLegend,) for EV depletion according to manufacturer’s instructions. Antibody-coupled beads were then incubated with concentrated MSC-CM (1mL) and applied to a magnetic column overnight (4°C). Depleted MSC-CM was then aspirated from each column. Depletion of TGF**-**β1 and sTNFR1 was validated by ELISA according to manufacturer’s instructions (R&D Systems, Minneapolis, MN, USA) (Supplementary Figure 2). Quantification of EVs with or without EV depletion was performed using imaging flow cytometry as previously described ^6^.

**Co-culture of PBMCs with depleted MSC-CM**

Cryopreserved PBMCs from healthy controls (n=3) and patient PBMC (n=3) were thawed, washed (450 x g, 10 mins) and resuspended at 2x10^6^ cells/mL in 10% FCS/RPMI. Cells were seeded into a 96-well round bottom tissue culture plate (200,000 cells/well). Cultures were set up in the absence of any stimulant (unstimulated), or with the addition of 0.1μg/mL lipopolysaccharide (LPS; Sigma-Aldrich, Castle Hill, NSW, AU). Cultures were prepared in a final volume of 200μL, containing 100μL of cells with 100μL of MSC-CM treated with anti-CD9/63/81, anti-TGF-β1, anti-TNFR or isotype control IgG. Final cultures were incubated at 37°C for 24 hours (5% CO2). Culture supernatants (150μL) were then collected and stored at -80°C until further use. Quantification of IL-8 in culture supernatants were performed by ELISA according to manufacturer’s instructions (BD Biosciences, San Jose, CA, USA).

**References**

1. Herrmann R, Sturm M, Shaw K, et al. Mesenchymal stromal cell therapy for steroid-refractory acute and chronic graft versus host disease: a phase 1 study. *Int J Hematol* 2012;95(2):182-8. doi: 10.1007/s12185-011-0989-2 [doi] [published Online First: 2011/12/21]

2. Johnson WE, Li C, Rabinovic A. Adjusting batch effects in microarray expression data using empirical Bayes methods. *Biostatistics* 2006;8(1):118-27. doi: 10.1093/biostatistics/kxj037

3. Armitage J, Tan D, Troedson R, et al. Mesenchymal stromal cell infusion modulates systemic immunological responses in stable COPD patients: A phase I pilot study. *European Respiratory Journal* 2018:1702369. doi: 10.1183/13993003.02369-2017

4. Dominici M, Le Blanc K, Mueller I, et al. Minimal criteria for defining multipotent mesenchymal stromal cells. The International Society for Cellular Therapy position statement. *Cytotherapy* 2006;8(4):315-17. doi: <http://dx.doi.org/10.1080/14653240600855905>

5. Forbes GM, Sturm MJ, Leong RW, et al. A phase 2 study of allogeneic mesenchymal stromal cells for luminal Crohn's disease refractory to biologic therapy. *Clin Gastroenterol Hepatol* 2014;12(1):64-71. doi: S1542-3565(13)01033-1 [pii]

10.1016/j.cgh.2013.06.021 [doi] [published Online First: 2013/07/23]

6. Armitage JD, Tan DBA, Cha L, et al. A standardised protocol for the evaluation of small extracellular vesicles in plasma by imaging flow cytometry. *J Immunol Methods* 2019;468:61-66. doi: 10.1016/j.jim.2019.03.006 [published Online First: 2019/03/20]

**Figure Captions**

**Supplementary Figure 1. Setup of the WGCNA pipeline.** To select the appropriate soft threshold, β is assigned values between 1-20, and adjacency matrices are generated for each soft threshold. A linear regression representing the log10(k) versus log10(p(k)) whereby p(k) is the proportions of genes with connectivity k, is calculated for each soft threshold. The linear regression R^2^ for each β value is shown (A) and the lowest β value to give an R^2^ of >0.85 (red line) is generally considered to be the appropriate soft threshold for biological networks. Based on these criteria a β=9 was selected, which satisfies scale-free topology and shows an exponential decay relationship between number of genes and their respective connectivity (B-C). A 1-TOM plot represents every gene in the analysis in an nxn matrix. The heatmap visualises the topological overlap between genes (yellow = low overlap, dark orange = high overlap). Cluster dendrograms on the left and top of the heatmap depict the module assignment (D), generating 6 co-expressing gene modules that are colour-designated (E).

**Supplementary Figure 2. Co-culture of PBMCs with MSC-CM depleted for EVs, TGF-β1 or sTNFR1.** Levels of EVs (A) TGF-β1 (B) and sTNFR1 (C) were measured in MSC-CM following magnetic depletion of each respective mediator (dEV, dTGF-β1 or dTNFR1) versus depletion with isotype control antibody. Measurement of IL-8 production (normalised against isotype control cultures, set at 100%) by unstimulated PBMC (D) or LPS-stimulated PBMC (E) following co-culture with depleted MSC-CM. Data points above the red dotted line (100%) indicate an increase in IL-8 production following co-culture with depleted MSC-CM.
